# One step at a time in investigating relationships between self-directed behaviours and parasitological, social and environmental variables

**DOI:** 10.1098/rsos.170461

**Published:** 2017-08-09

**Authors:** Julie Duboscq, Valéria Romano, Cédric Sueur, Andrew J. J. MacIntosh

**Affiliations:** 1 Kyoto University Primate Research Institute, Kanrin 41-2, 484-8506 Inuyama, Japan; 2 CNRS, IPHC UMR 7178, Université de Strasbourg, 23 rue Becquerel, 67087 Strasbourg Cedex 2, France

We thank Norscia and Palagi for their insightful commentary on our article ‘Scratch that itch: revisiting links between self-directed behaviour and parasitological, social and environmental factors in a free-ranging primate’ [1]. We welcome such discussion because we think, as the authors themselves point out at the end of their commentary, that research needs to continue in this area. In general, we agree that different stressors may act at different time frames in triggering self-directed behaviours. As rightly pointed out by Norscia and Palagi, our analysis did not take into account the different time frames that would allow for separating the effects of acute and chronic stressors on self-directed behaviours. At the level of a behavioural observation of 15 min, we instead investigated whether the occurrence of scratching and self-grooming was linked to various factors such as lice load, social activities, neighbours in proximity and environmental conditions, together and/or separately. Our study was correlational and we, therefore, avoided claims of causality, although we did address potential causal mechanisms in the discussion.

That said, we would nonetheless like to respond to several points made by Norscia and Palagi. First, one of the main points of our study was to highlight biases in the investigation of certain research hypotheses, such as those involving self-directed behaviours. Studies in primatology have often, if perhaps inadvertently, assumed that the primary drivers of self-directed behaviour (SDB) are social, with parasite or abiotic factors being secondary. Norscia and Palagi nonetheless state that ‘(…) the association between self-directed behaviours, and particularly scratching, with social, environmental and parasitological factors can be considered as more than just a hypothesis. Once established that the different factors are not alternative and that their relationship with scratching has been demonstrated, it is worth focusing on the role that each factor can have in relation to the time scale’ (p. 2). We would fully agree with the logic here if the premise were true. While the association between parasitological factors and self-directed behaviour is extremely well-established in ungulates and birds [2–16], with also some evidence in insects [17–19], it has received surprisingly little attention in non-human primate research. Despite the fact that numerous earlier studies on the functional significance of self- and social grooming did mention the removal of ectoparasites [20–23], it has sometimes been dismissed groundlessly or ignored altogether in more recent studies [24–27]. There is no *a priori* or *a posteriori* reason to assume that what affects ungulates or birds does not affect primates when the system under study, in this case the ectoparasite–host system, is more or less identical. Along these lines, our study was an attempt to test multiple hypotheses simultaneously and objectively using the same comprehensive dataset. A multivariate approach perhaps provides the best opportunity to draw out the key factors influencing behaviour, and thereby contribute to advancing the field. All speculation aside, our study reveals that, among the candidate set of hypotheses tested (formulated as statistical models), parasite factors appear to best explain the occurrence of scratching, while parasite and social factors appear to do so for self-grooming. If future work can now tease out the impacts of these and other factors at distinct time scales while also accounting for alternative explanations, such work would be most welcome indeed.

Second, in our study, at the level of the aggregate dataset, the hypotheses put forward are indeed non-mutually exclusive in explaining general SDB patterns, as noted by Norscia and Palagi. However, at the level of an individual SDB event, each of the hypotheses is more likely to explain the behaviour independently than in concert, though we also acknowledge the possibility of additive or even synergistic effects here; note that our statistical models for self-grooming suggested that such additive effects were likely. Regardless, a single SDB may be caused by *x*, *y* or *z*, but seems less likely to occur because of all three simultaneously, so the use of the term ‘alternative’ is not necessarily incorrect. That said, contrary to the assertions of Norscia and Palagi, this does not imply that some relationships are secondary to others. We think this distinction is meaningless, and that is why we took an integrative approach in the first place. If we did not make that point clear enough in the original manuscript, then we reiterate it here.

Third, some of the arguments put forth by Norsica and Palagi involve generalizations that may not in fact be entirely supported. Essential facts concerning primates—and to some extent time scales—are omitted in their commentary. For instance, several studies have already demonstrated quite unambiguously that body parts estimated to have many louse eggs are generally inaccessible, cannot be self-groomed, and are socially groomed longer than other body parts [8,10,15,24,28,29]. Furthermore, lice loads estimated from nit-picking gestures during grooming were recently shown to vary seasonally in Japanese macaques [30], and variation in nit-picking activity during grooming, or louse-egg feeding, has been shown to influence grooming duration, frequency and reciprocity [28]. The findings in [29] and [28] are especially important, not only because they align what we know about primates with what we know about birds and ungulates, but also because they relate to the extent to which ectoparasites can mediate social interactions, a hypothesis that is rarely acknowledged in primate studies (e.g. [30,31]). The facts that treating animals against lice decreases grooming activity and that preventing animals from grooming or self-grooming dramatically increases ectoparasite load [8,15] speak volumes in favour of investigating the links between ectoparasites and SDB, in addition to further social processes also linked to hygienic practices, regardless of time scale. So, we would argue that before dissecting *when* or under what set of conditions a certain event is likely to occur, we need to first ensure that the event and these other conditions are indeed generally related. From our perspective, such an investigation has never been fully realized in taxa as socially complex as primates, and we therefore feel the approach taken in our original article is justified.

Finally, Norscia and Palagi state that ‘The variation observed between time *t*_0_ and *t*_1_ cannot be linked to parasitological factors if the load is not significantly different between *t*_0_ and *t*_1_. There is no reason to believe that, in the absence of any other additional perturbing factor, the ectoparasite load varies significantly in the minutes immediately preceding and following the stressful event.’ (p. 2) While this statement belies a lack of knowledge about louse behaviour (i.e. temporal patterns in feeding behaviour), to their credit the authors do later add that ‘It may be questioned that in the short term a change in the parasite activity (e.g. in response to temperature, humidity or even solar radiation [18–22]), and not in the load, could possibly cause an increase in scratching levels. However, this aspect was not tested in Duboscq *et al*. [1].’ (p. 2) Louse-induced itch could indeed depend in the long term on louse load and in the short term on louse activity. A sudden change in a multitude of parameters might impact louse load and/or activity quickly if it creates disturbances in the hair/pelage/skin of the animals that constitutes the environment of the parasite. Some studies have shown that rabbit fleas respond to oestrogen blood concentration and adapt their reproductive activity to the reproductive activity of their host [32,33]. The variation in self-directed behaviours between *t*_0_ and *t*_1_ could therefore be linked to parasitological factors at time scales of minutes or even seconds, although we admit that we have no information about whether louse activity is likely to vary ‘in the minutes immediately preceding and following the stressful event’ (p. 2). Again, we have no intention here of asserting that all SDB events are related to lice, but the effect of variation in ectoparasite load and activity across time scales should be investigated in the future, and we think our study constitutes a step forward in that direction.

In conclusion, while we agree with most of the comments provided by Norscia and Palagi, we highlight that the aims of our study were not so much to exclude the role of social stressors in the production of SDB but instead to put SDB into the broader ecological framework under which they evolved. Like Norscia and Palagi, we look forward to future studies taking an integrative view of self-directed behaviours, accounting for various factors at different time scales in order to gain further insights into why animals scratch that itch.

## Data accessibility

This article has no additional data.
